# Core decompression assisted by multi-functional minimally invasive instruments for the treatment of early osteonecrosis of the femoral head

**DOI:** 10.1038/s41598-025-90551-w

**Published:** 2025-02-19

**Authors:** Daizhu Yuan, Zhanyu Wu, Yuhu Zhou, Jianxiang Teng, Qiuhan Chen, Chuan Ye

**Affiliations:** 1 Department of Orthopaedics and Sport Medicine, The Affiliated Hospital of Guizhou Medical University, Guiyang, 550004 China; 2 Center for Tissue Engineering and Stem Cells, Guizhou Medical University, Guiyang, 550004 China

**Keywords:** Medical research, Translational research

## Abstract

Core decompression is a common method for treating early osteonecrosis of the femoral head (ONFH). However, the surgical procedure is cumbersome due to the lack of appropriate surgical instruments. This study aims to modify surgical instruments to improve surgery efficiency. A total of 28 patients with early ONFH treated with the core decompression were enrolled. 13 cases were treated with new instruments and the other 15 cases were treated with the traditional methods. The convenience of the new instruments was evaluated by comparing evaluation indicators. The multi-functional instruments reduced the number of fluoroscopy, shorted the operation time, improved the delivery efficiency, reduced the intraoperative blood loss, and reduced the surgical incision compared with the traditional method (*p* < 0.05). The new instruments removed the healthy bone of the femoral head and neck for reuse, the overall hospitalization cost was lower, and patient satisfaction was higher (*p* < 0.05). In the postoperative follow-up, the VAS was lower and Harris score was higher compared with the traditional group (*p* < 0.05). The multi-functional instruments can achieve the advantages of accurate positioning of the necrotic area, removed and reused healthy bone, effective expanded decompression, and efficient implant delivery, which is the effective instrument for the early ONFH.

## Introduction

Osteonecrosis of the femoral head (ONFH) is a common refractory disease in clinical practice, largely caused by hip trauma, immunosuppressant abuse, and long-term alcohol abuse^1^. As the disease continues to progress, progressive collapse of the femoral head can occur in advanced stages, leading to the destruction of hip function and seriously affecting the quality of life^2^. Therefore, it is important to perform the hip preservation before the early stage of femoral head collapse in the early stage^3^.

At present, core decompression or decompression combined with other methods are mainly used for the treatment of early ONFH, such as stem cell transplantation^4^, platelet-rich plasma^5^, tantalum rod implantation^6^, and bone grafting^7^. However, there are shortcomings due to the lack of special surgical instruments: (1) Difficult location of necrotic areas: Orthopedic surgeons need to adjust the positioning angle of the proximal femur (anteversion angle and neck-shaft angle) several times and locate the necrotic area during the surgery, which leads to time-consuming and large intraoperative X-ray radiation. Many scholars have studied the positioning technology, including 3D printing guide plate technology^8^, MRI-assisted localization technique^9^, and robot-assisted technology^10^, which can achieve accurate positioning of the necrotic area. However, the 3D printed guide plate is a personalized consumable, which cannot be reused and increases the extra cost of the patient. Moreover, the incision needs to be enlarged to meet the guide plate adhering to the bone surface. The MRI-assisted localization technique is cumbersome and increases the operation time. Instruments for robot-assisted navigation are expensive and difficult to make widely available in routine hospitals. (2) Destruction and waste of healthy bone: The hollow drill was applied to break through the necrotic hardening zone and decompress after the location of the necrotic area of the femoral head^11^. However, the process of core decompression will cause the complete destruction and waste of healthy bone in the femoral head and neck. Studies have shown that the bone mud generated during drilling decompression can be collected to refill the necrotic area for reuse. However, it is easy to lose and needs to be mixed with other graft materials^12^, which increases the cost of consumables. (3) Limitation of effective expanded decompression: Expanded decompression of the necrotic area is required after core drilling decompression for patients with a large area of necrosis^11^. However, it is difficult to effectively expand decompression around the necrotic lesion through the lateral wall of the trochanteric due to the long and narrow bone tunnel^13^. Studies have shown that the application of enlarged reamers can carry out large-scale lesion clearance and decompression in the necrotic area^14,15^. However, it can lead to a decrease in the mechanical strength of local bone structure and increase the risk of femoral head collapse. (4) Low efficiency of implant delivery: In order to ensure the clinical effect, the related grafts, such as stem cells, platelet-rich plasma, and bone grafts, were delivered to the necrotic area through the narrow bone tunnel on the lateral wall of the trochanter after core decompression^4–7^. However, the process of graft delivery is time-consuming and laborious due to the interference of rich soft tissue in the proximal femur and the narrow bone tunnel^16,17^. Researchers have made a device for bone marrow stem cell transplantation to assist core decompression through 3D printing technology^18^. However, the device requires customization, increases the cost, and there are doubts about whether delivery of other graft materials can be performed.

The above shortcomings lead to prolonged operation time, large trauma, and low experience of doctors, and it is difficult to promote this surgical method in clinical practice. Therefore, the purpose of this study is to develop a multi-functional surgical instrument for core decompression, including accurate location of the necrotic area, reuse of healthy bone, effective expanded decompression, and efficient graft delivery. The new surgical instruments make the operation simple and feasible, and further shorten the operation time, reduce the number of intraoperative X-ray radiation exposure, and reduce surgical trauma.

## Materials and methods

This study conforms to the provisions of the Declaration of Helsinki and has been reviewed and approved by the Institutional Review Board of the Affiliated Hospital of Guizhou Medical University (No.300 in 2023). The informed consent has been obtained from involving participants.

### Introduction to the multi-functional minimally invasive instruments

**Fig. 1:**
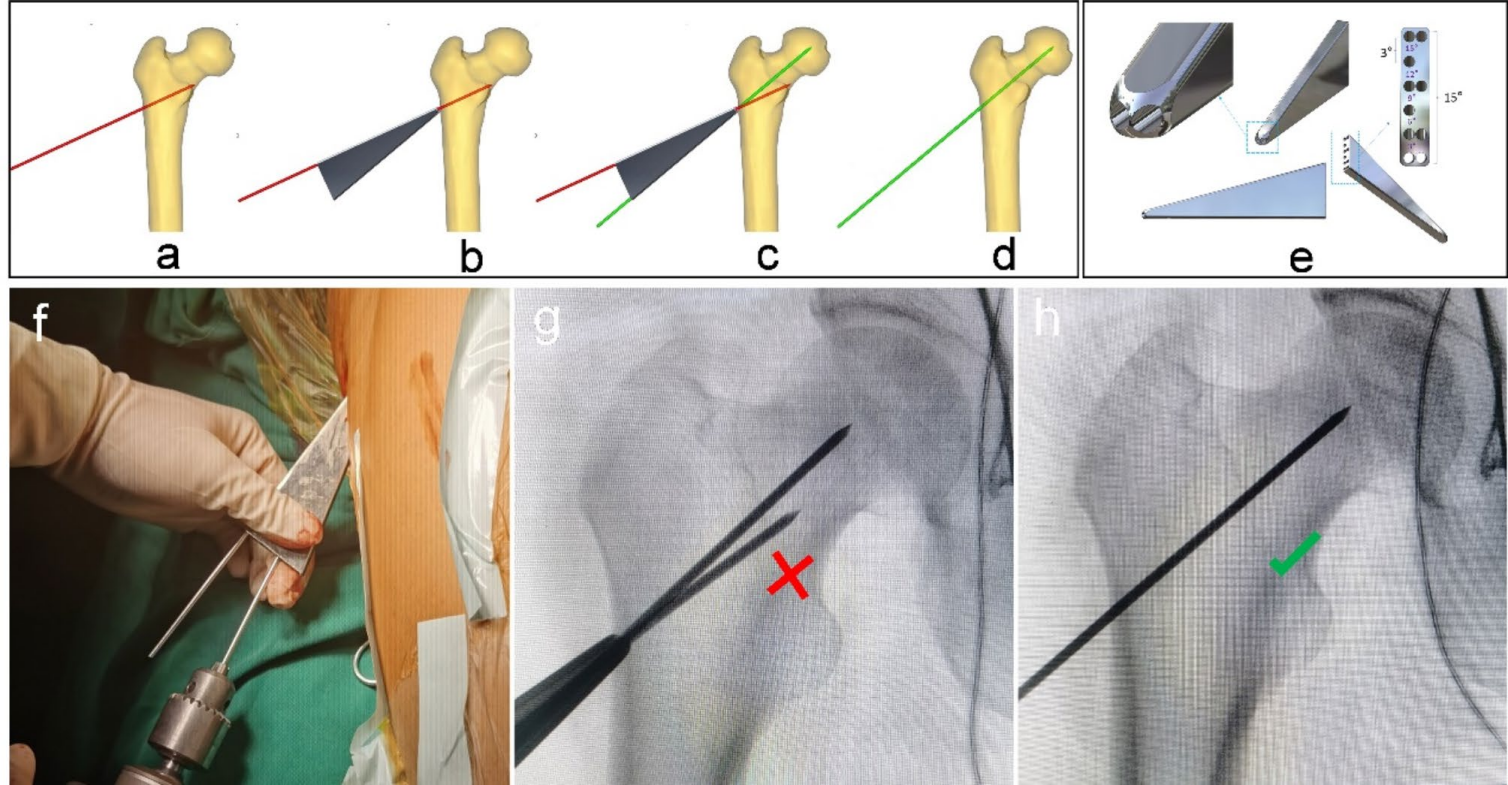
The accurate positioning device accurately located the necrotic parts by the adjustment of femoral neck-shaft Angle. (**a**) The Kirschner wire was inserted into the femoral head and neck and positioned inaccurately; (**b**) The device contacted the bone surface by connecting the Kirschner wire; (**c**) An additional Kirschner wire was inserted according to the adjustment of angle; (**d**) The ideal position of the Kirschner wire to be inserted into the necrotic parts of the femoral head; (**e**) The three-dimensional (3D) model of the device; (**f**) Intraoperative use of the device; (**g**) Not ideal localization of neck-shaft Angle of Kirschner wire, using the accurate positioning device and quantitative adjustment of femoral neck-shaft Angle under X-ray fluoroscopy; (**h**) Ideal localization of neck-shaft Angle of Kirschner wire.

**Fig. 2:**
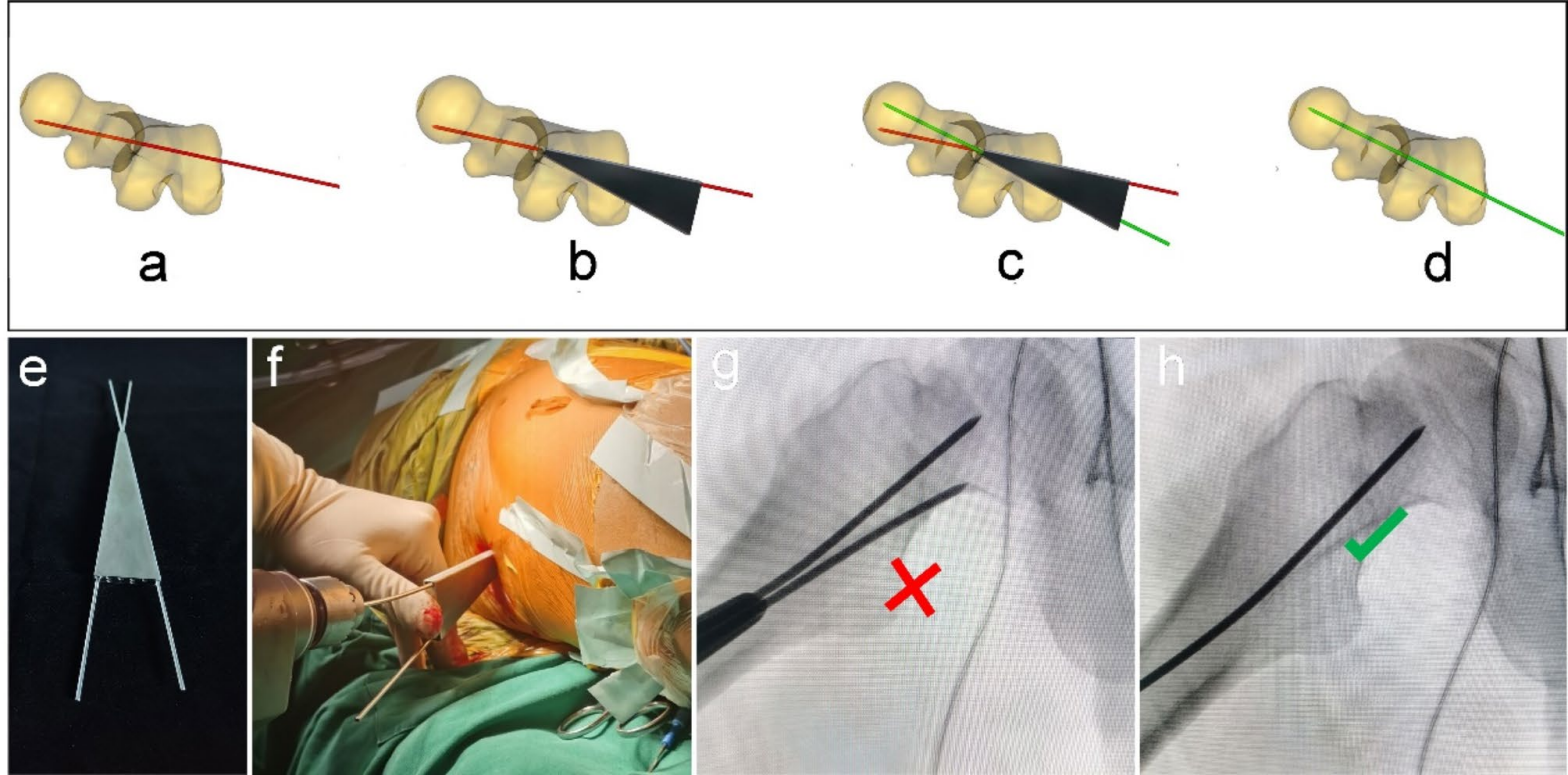
The accurate positioning device accurately located the necrotic parts by the adjustment of femoral anteversion Angle. (**a**) The Kirschner wire was inserted into the femoral head and neck and positioned inaccurately; (**b**) The device contacted the bone surface by connecting the Kirschner wire; (**c**) An additional Kirschner wire was inserted according to the adjustment of angle; (**d**) The ideal position of the Kirschner wire to be inserted into the necrotic parts of the femoral head; (**e**) The general appearance of the device; (**f**) Intraoperative use of the device; (**g**) Not ideal localization of femoral anteversion Angle of Kirschner wire, using the device and quantitative adjustment of femoral anteversion Angle under X-ray fluoroscopy; (**h**) Ideal localization of femoral anteversion Angle of Kirschner wire.

**Fig. 3:**
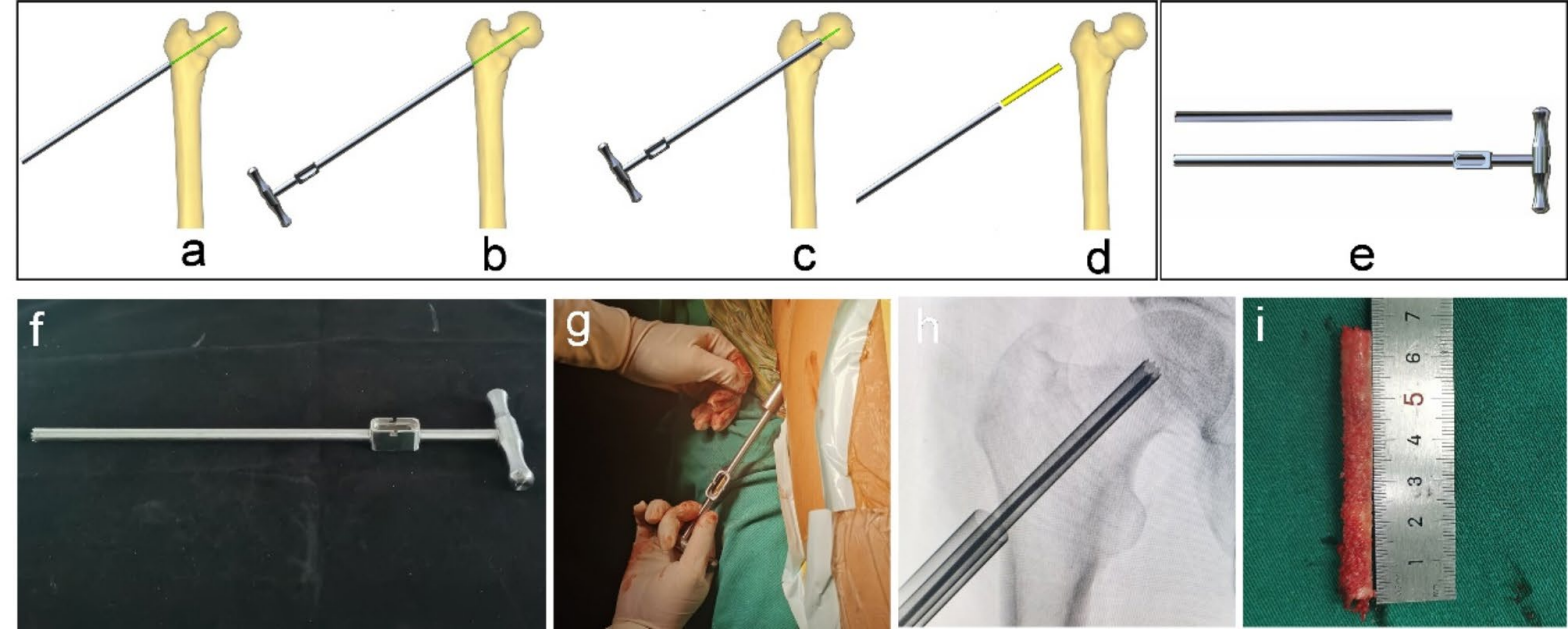
Device of bone harvesting and decompression combined both gaining healthy bone and decompression by the removal of necrotic parts. (**a**) The hollow guide device connected the Kirschner wire that had accurately located the necrotic parts using the accurate positioning device; (**b**-**c**) The device of bone harvesting and decompression connected the hollow guide device, rotated the T-handle handle of the device, and advanced to the necrotic parts; (**d**) Both gaining healthy bone and decompression by the accuracy of removal of necrotic parts; (**e**) The 3D model of the device; (**f**) The general appearance of the device; (**g**) Intraoperative use of the device; (**h**) The device rotated and advanced to the necrotic parts; (**i**) The healthy bone of femoral head and neck was obtained through pulling out the device while decompression was achieved by the accuracy of removal of necrotic parts.

**Fig. 4:**
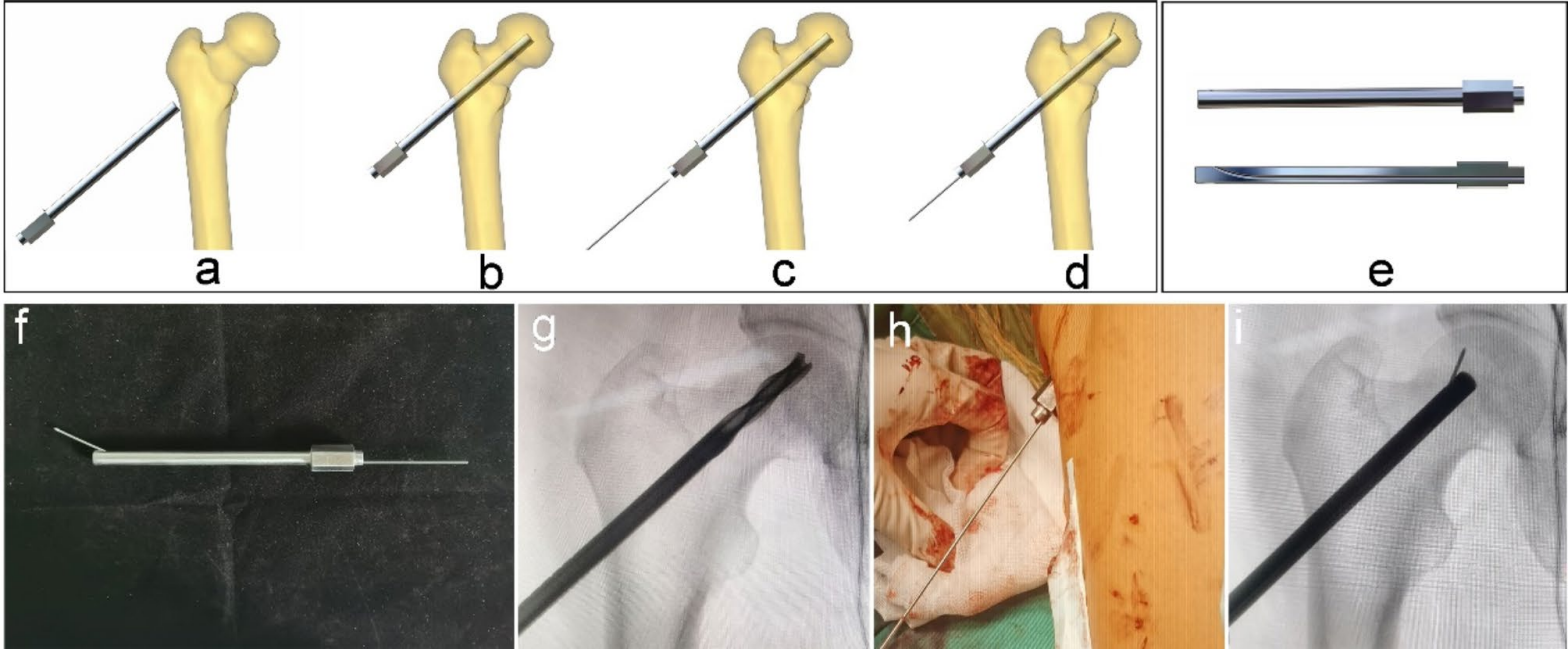
Expanded decompressor enlarged the decompression range of the necrotic parts by punching small holes. (**a**-**b**) The expanded decompressor inserted the bone tunnel that formed by the device of bone harvesting and decompression and reached the necrotic area; (**c**-**d**) The device connected the small Kirschner wire and enlarged the decompression range of the necrotic parts by punching small holes; (**e**) The 3D model of the device; (**f**) The general appearance of the device; (**g**) Removing the remaining sclerotic necrotic bone with a hollow drill before using the expanded decompressor; (**h**) Intraoperative use of the device; (**i**) Punching small holes outward through the Kirschner wire and enlarging the decompression range of the necrotic area.

**Fig. 5:**
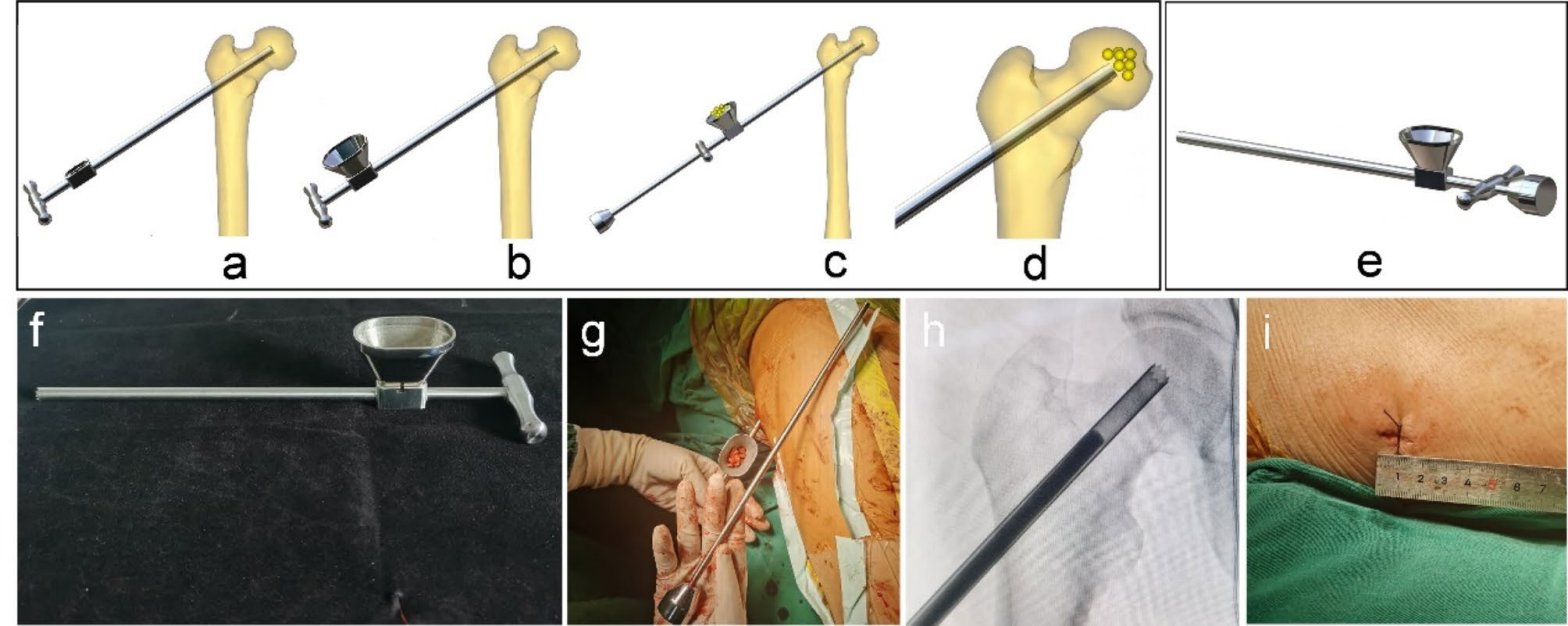
Implant delivery device improved the efficiency of implant delivery. (**a**) The device of bone harvesting and decompression that served as the delivery channel Inserted the bone tunnel to reach the necrotic area; (**b**) Connecting to the graft infundibulum; (**c**-**d**) Placing the crushed bone that was the healthy bone of femoral head and neck into the graft infundibulum and pushing crushed bone through the top rod to the necrotic lesion area; (**e**) The 3D model of the device; (**f**) The general appearance of the device; (**g**) Intraoperative use of the device; (**h**) Pushing crushed bone through the top rod to the necrotic lesion area; (**i**) Length of surgical incision.

The multi-functional instruments (Biocare Biotechnology Co. LTD, China, Zhejiang) included the four devices (accurate positioning device, the device of bone harvesting and decompression, the expanded decompressor, and the implant delivery device) and four functions. (1) Accurate positioning device: Accurate position of necrotic parts was achieved by the respective adjustment of femoral neck-shaft Angle and anteversion Angle, and the adjustment of the two angles does not interfere with each other (Figs. 1 and 2). (2) Device of bone harvesting and decompression: The device connected the hollow guide device and the Kirschner wire that had accurately located the necrotic parts using the accurate positioning device, then rotated the T-handle handle of the device, and advanced to the necrotic parts. The healthy bone of femoral head and neck could be obtained through pulling out the device while decompression was achieved by the accuracy of removal of necrotic parts (Fig. 3). (3) Expanded decompressor: Performing effective expanded decompression of the necrotic area through a narrow bone tunnel (Fig. 4). (4) Implant delivery device: Reaching necrotic area and performing efficient delivery of implants by avoiding soft tissue interference (Fig. 5).

### Basic information of patients

28 patients (17 males and 11 females, aged from 21 to 55 years) with early ONFH who underwent unilateral core decompression participated in this study from November 2020 to June 2023. The traditional method group: 15 cases were treated with traditional core decompression combined with bone grafting, including 9 males and 6 females, 6 cases on the left side and 9 cases on the right side, 4 cases in Association Research Circulation Osseous (ARCO) stage I and 11 cases in ARCO stage II. The new method group: 13 cases were treated with modified techniques and instruments, including 8 males and 5 females, with 5 cases on the left side and 8 cases on the right side, 4 cases in ARCO stage I and 9 cases in ARCO stage II. There was no significant difference in preoperative count data such as gender, sides of hip joint, ARCO stage between the two groups (Table 1).

**Table 1:** Demographic characteristics of patients.

Parameter	Traditional Method	New Method	*p* Value
Sex (men/women)	9/6	8/5	> 0.05
Age, Years	41.5 ± 3.7	43.0 ± 4.9	> 0.05
ARCO stage (I/II stage)	4/11	4/9	> 0.05
Sides of hip joint (left/ right)	6/9	5/8	> 0.05
Preoperative Harris score	77 ± 2.8	75 ± 5.2	> 0.05

*ARCO* association research circulation osseous.

### Surgical techniques and procedures

The new multi-functional instrument requires only four steps to complete the surgical procedure of core decompression, including accurate position, bone harvesting and decompression, expanded decompression, and efficient delivery. (1) Accurate position: A 2.5 mm Kirschner wire was placed into the necrotic area through the lateral wall of the trochanter, and the initial localization was performed by X-ray fluoroscopy. The soft tissue was separated through a small incision and the accurate positioning device was connected to the Kirschner wire to reach the bone surface. Another Kirschner wire was connected to the device to accurately adjust the neck-shaft Angle and anteversion Angle (Figs. 1 and 2). (2) Bone harvesting and decompression: The device of bone harvesting and decompression was inserted and gradually rotated and advanced to the necrotic area after using the soft tissue protection device. When the device reached the necrotic area, it was pulled out to gaining healthy bone and decompression by the removal of necrotic parts, and crushed the healthy bone for later use (Fig. 3). (3) Expanded decompression: The expanded decompressor was inserted through the bone tunnel to reach the necrotic area after removing the remaining sclerotic necrotic bone with a hollow drill. Then, the expanded decompression of the necrotic area was achieved by expanding the drill hole outward through a 1.5 mm Kirschner wire (Fig. 4). (4) Efficient delivery: The device of bone harvesting and decompression was inserted into the necrotic area through a bone tunnel and connected to the graft infundibulum. The crushed bone was placed into the graft infundibulum and pushed through the top rod to the necrotic lesion area (Fig. 5).

### Evaluation indicator

The evaluation indicators included operative outcomes, postoperative outcomes, and clinical outcomes of the follow-up. The operative and postoperative outcomes included number of fluoroscopy, operation time, intraoperative blood loss, length of incision, hospitalization costs, number of surgeons, and patient satisfaction. The clinical outcomes of the follow-up included Harris score, VAS score, postoperative complications and MRI evaluation. The repair of necrotic area was evaluated by MRI based on the surface area ratio method^19^.

### Statistical analysis

SPSS 24.0 (IBM Inc, Armonk, New York, USA) software was used for statistical analysis. Data extracted were tested for normality using the Shapiro–Wilk test. Normally distributed measurement data was expressed as mean ± standard deviation, and inter-group comparisons was conducted using the independent samples t-test. Data that did not conform to a normal distribution was expressed as M (Q1, Q3), and inter-group comparisons utilized non-parametric rank sum tests. The comparison of count data, including gender, sides of hip joint, and ARCO stage, employed Fisher’s exact probability method because the sample size of this study was less than 40. *p <* 0.05 was considered to be statistically significant.

## Results

### Operative and postoperative outcomes

The application of accurate positioning instruments in the new method group could achieve accurate adjustment of neck-shaft Angle and anteversion Angle of Kirschner wire during operation, and reduce the number of intraoperative X-ray fluoroscopy (3 ± 1.07 vs. 7 ± 1.58, *p* < 0.01) (Table 2). Moreover, the length of the surgical incision was smaller (1.7 ± 0.21 cm vs. 6.5 ± 1.34 cm, *p* < 0.01) than that of the traditional positioning method due to the conical design of the accurate positioning device (Table 2). With the assistance of multifunctional surgical instruments and the improvement of surgical procedures, core decompression can be completed in only four steps, which can shorten the overall operation time (34 ± 4.22 min vs. 65 ± 7.85 min, *p* < 0.01) and reduce intraoperative blood loss (9 ± 1.80 ml vs. 35 ± 4.14ml, *p* < 0.01) compared with the traditional core decompression method (Table 2). The device of bone harvesting and decompression could can decompress the necrotic area and take out the healthy bone inside the femoral head and neck for reuse, which could reduce the overall hospitalization costs compared with the traditional method group (*p* < 0.01) (Table 2). The operation of multifunctional surgical instruments required only one person to perform the operation, and occasionally an assistant was needed to help internal rotation of the lower limbs to facilitate the placement of Kirschner wires, which can save the number of surgeons attending the procedure compared with the traditional method (*p* < 0.01) (Table 3). Scores of the patient satisfaction was higher (*p* < 0.01) (Table 2) than that of the traditional method group due to the advantages of smaller surgical incision, lower hospitalization cost, and less intraoperative radiation.

**Table 2:** Operative and postoperative outcomes (mean ± standard).

Evaluation Indicators	Traditional Method (*n* = 15)	New Method (*n* = 13)	*p* Value
Number of fluoroscopy	7 ± 1.58	3 ± 1.07	< 0.01
Operation time (min)	65 ± 7.85	34 ± 4.22	< 0.01
Intraoperative blood loss (ml)	35 ± 4.14	9 ± 1.80	< 0.01
Length of incision (cm)	6.5 ± 1.34	1.7 ± 0.24	< 0.01
Hospitalization costs (10,000 ¥)	2.1 ± 0.30	1.2 ± 0.21	< 0.01
Patient Satisfaction	73 ± 4.22	91 ± 4.65	< 0.01
Harris score			
1 Weeks after operation	73 ± 6.60	74 ± 5.30	0.534
3 Months after operation	76 ± 4.54	83 ± 6.89	< 0.05
6 Months after operation	79 ± 4.52	89 ± 2.96	< 0.01
9 Months after operation	82 ± 2.43	93 ± 3.83	< 0.01
12 Months after operation	83 ± 3.74	94 ± 2.03	< 0.01

**Table 3:** Operative and postoperative outcomes M (Q1, Q3).

Evaluation Indicators	Traditional Method (*n* = 15)	New Method (*n* = 13)	Z	*p* Value
Number of surgeons	3 (2, 4)	1 (1, 2)	-4.198	< 0.01
VAS score				
1 Week after operation	5 (5, 6)	4 (4, 5)	-2.356	< 0.05
3 Months after operation	4 (4, 5)	3 (2, 3)	-3.614	< 0.01
6 Months after operation	4 (3, 5)	2 (2, 3)	-4.089	< 0.01
9 Months after Operation	4 (3, 4)	2 (1.5, 3)	-4.157	< 0.01
12 Months after Operation	4 (3, 4)	2 (1.5, 3)	-3.767	< 0.01

*VAS* visual analogue scale.

### Clinical outcomes of the follow-up

**Fig. 6:**
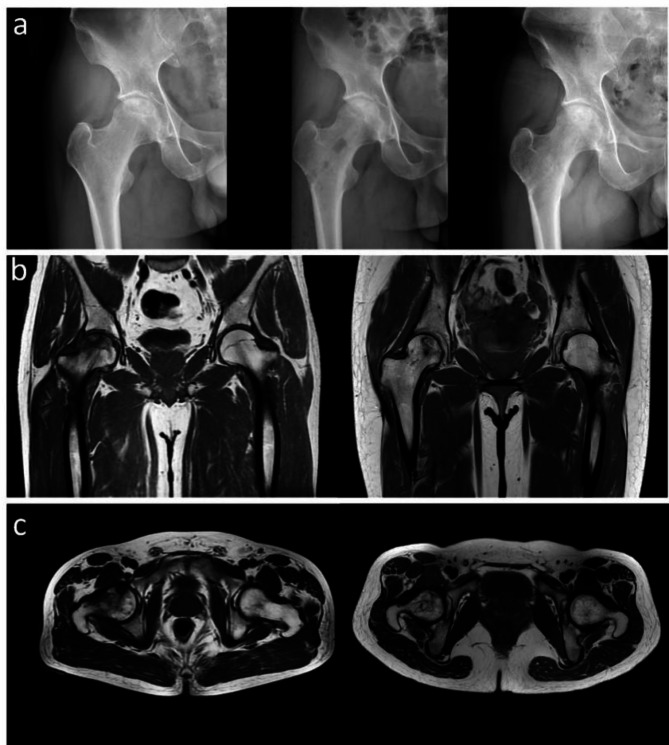
(**a**) X-rays of pre-operation, 3months after operation, and 9 months after operation; (**b**) Coronal MRI of pre-operation and 9 months after operation; (**c**) Cross-sectional MRI pre-operation and 9 months after operation.

There was no significant difference in Harris score between the two groups at 1 week after operation (74 ± 5.3 vs. 73 ± 6.6, *p* > 0.05). However, at 3, 6, 9 and 12 months after operation, the Harris hip score of the new method group was higher than that of the traditional method group (*p* < 0.05) (Table 2). As shown in Table 2, the Harris scores of the two groups showed a gradual upward trend during the postoperative follow-up, and the new device method group was more obvious than the traditional method group. The pain symptoms of the new device method group were relieved and the VAS score was lower, and the difference was statistically significant compared with the traditional method group (*p* < 0.01) (Table 3). The VAS scores of the two groups showed a gradual downward trend during the postoperative follow-up, and the new device method group was more obvious than the traditional method group (Table 3). There were no associated complications such as delayed wound healing, infection, fracture, instrument trouble, nerve injury and vascular injury in the new method group. There were no infection, fracture and other complications in the traditional group, but one patient had delayed wound healing. As shown in Fig. 6, at 3 months after core decompression, X-ray showed that the bone tunnel was partially filled with bone in the new device method group, all the bone tunnels were filled with bone by 9 months after operation, and MRI showed that the area of femoral head necrosis was less than that before operation at 9 months after operation.

## Discussion

The minimally invasive multifunctional surgical instrument of this study for core decompression improve the surgical efficiency of orthopedic surgeons, which can achieve the advantages of accurate positioning of the necrotic area, the healthy bone was removed and reused, effective expanded decompression and efficient delivery of implants in the necrotic area.

Inaccurate localization of the necrotic area may lead to incomplete decompression of the lesion area or excessive destruction of normal bone tissue, leading to decreased mechanical properties^20,21^. The traditional positioning method requires multiple intraoperative X-ray fluoroscopy to obtain the anteroposterior and lateral images of the hip joint to adjust the neck-shaft Angle and the anteversion Angle of the Kirschner wire positioning direction^22^, which increases the intraoperative radiation dose and prolongs the overall operation time. In addition to the interference of rich soft tissue in the proximal femur, the main reason for the difficulty of traditional positioning is the lack of positioning quantitative reference, which leads to the operation of each positioning is subjective experience, without quantitative changes based on the initial positioning situation, resulting in each positioning is a new attempt. As shown in Fig. 1, the accurate positioning device designed in this study could locate the necrotic area through the quantitative reference of Angle parameters marked on the new device, and each new re-positioning can be closer to the ideal positioning position. Moreover, the new instrument can realize the adjustment of neck-shaft Angle and anteversion Angle respectively, without interference with each other. As shown in Table 2, compared with the traditional method group, the new method group could reduce the number of intraoperative X-ray fluoroscopy, thereby reducing the radiation to patients and doctors. Some orthopedic surgeons even develop radiation-induced diseases due to the need for intraoperative X-ray fluoroscopy to assist in the evaluation of fracture reduction^23,24^. As shown in Fig. 5; Table 2, the new method group had a smaller surgical incision than the traditional method group, which truly achieved the purpose of minimally invasive surgery. Moreover, a smaller incision can reduce postoperative pain and improve the patient’s hospital experience. With the improvement of medical level, the concept of minimally invasive surgery is more easily accepted by patients and doctors, and minimally invasive surgery is the future direction of surgery^25^. At present, minimally invasive surgery has been developing rapidly in the field of orthopedics^26,27^.

Bone grafting in the necrotic area after core decompression is an effective method for the treatment of early osteonecrosis of the femoral head^28^. Autologous bone is mainly harvested from iliac crest, and there are complications such as pain in the donor site, nerve injury, hematoma, and infection^29^. Allogeneic bone can be considered, but it is expensive and increases the overall cost of hospitalization^30^. The device developed in this study can decompress the necrotic area and extract the healthy bone from the femoral head neck for reuse. As shown in Fig. 3, approximately 7 cm of the structural bone column was successfully removed during the procedure, avoiding the disadvantages caused by traditional bone harvesting and highlighting the advantages of the device of bone harvesting and decompression.

The decompression range of traditional core decompression is the diameter of the core drill, and the decompression range is limited, which leads to the inability to open the sclerosis zone of the necrotic area and achieve the purpose of sufficient decompression, affecting the postoperative clinical efficacy^31^. Although the diameter of the core drill can be increased to expand the decompression range, it will destroy the local biomechanical stability and increase the risk of collapse and fracture^32^. The further expanded decompression of the necrotic area is difficult within the narrow bone tunnel. As can be seen from Fig. 4, the expanded decompressor in this study directly reaches the necrotic area through the bone tunnel, realizing the expanded decompression of the narrow bone tunnel and reducing the damage to the surrounding bone structure by using the fan-shaped radial guidance function inside the device. As can be seen from Table 2, the overall operation time of the new method group was shorter than that of the traditional method group, indicating that the whole operation steps and process of core decompression were improved and simplified. Only four steps of accurate positioning, bone decompression, expanded decompression and efficient delivery were needed to complete the operation, thereby shortening the overall operation time and benefiting the patients. The duration of surgery is related to the rate of postoperative incision infection, that is, the prolonged duration of surgery will lead to the incision being exposed to air for longer, and more opportunities for bacteria to contact the incision and increase the risk of infection^33^.

The multifunctional surgical instrument designed in this study can effectively assist the procedures for core decompression and simplify the surgical procedures. However, there are some limitations. First, this study was retrospective, lacking randomized controls and blinding, and involved a small sample size. a large-sample, multicenter, blinded randomized controlled trial is still required to further confirm the conclusions of our current study. Second, the use of allogeneic bone grafting in the traditional method group may affect the comparison of clinical efficacy between the two groups. Finally, the postoperative follow-up time was relatively short.

## Conclusion

The modified instruments and surgical procedures can achieve the advantages of accurate positioning of the necrotic area, the healthy bone was removed and reused, effective expanded decompression of the necrotic area and efficient implant delivery, which is the effective instruments for the treatment of early ONFH.

## Data Availability

The datasets used and analyzed during the current study are available from the corresponding author upon reasonable request.
